# S1P Lyase Regulates Intestinal Stem Cell Quiescence via Ki-67 and FOXO3

**DOI:** 10.3390/ijms22115682

**Published:** 2021-05-26

**Authors:** Anja Schwiebs, Farha Faqar-Uz-Zaman, Martina Herrero San Juan, Heinfried H. Radeke

**Affiliations:** Institute of General Pharmacology and Toxicology, Pharmazentrum Frankfurt/ZAFES, Hospital of the Goethe University, 60590 Frankfurt am Main, Germany; w.farha8@gmail.com (F.F.-U.-Z.); herrero@em.uni-frankfurt.de (M.H.S.J.)

**Keywords:** S1P lyase, colon cancer, cell cycle, quiescence, Ki-67, FOXO3, CDK2, AKT signaling, SGPL1 knockout cell line

## Abstract

Background: Reduction of the Sphingosine-1-phosphate (S1P) degrading enzyme S1P lyase 1 (SGPL1) initiates colorectal cancer progression with parallel loss of colon function in mice. We aimed to investigate the effect of SGPL1 knockout on the stem cell niche in these mice. Methods: We performed immunohistochemical and multi-fluorescence imaging on tissue sections of wildtype and SGPL1 knockout colons under disease conditions. Furthermore, we generated SGPL1 knockout DLD-1 cells (SGPL1^−/−M.Ex1^) using CRISPR/Cas9 and characterized cell cycle and AKT signaling pathway via Western blot, immunofluorescence, and FACS analysis. Results: SGPL1 knockout mice were absent of anti-Ki-67 staining in the stem cell niche under disease conditions. This was accompanied by an increase of the negative cell cycle regulator FOXO3 and attenuation of CDK2 activity. SGPL1^−/−M.Ex1^ cells show a similar FOXO3 increase but no arrest of proliferation, although we found a suppression of the PDK1/AKT signaling pathway, a prolonged G1-phase, and reduced stem cell markers. Conclusions: While already established colon cancer cells find escape mechanisms from cell cycle arrest, in vivo SGPL1 knockout in the colon stem cell niche during progression of colorectal cancer can contribute to cell cycle quiescence. Thus, we propose a new function of the S1P lyase 1 in stemness.

## 1. Introduction

Sphingolipids and their bioactive metabolites are involved in structural maintenance of cell membranes and mediate cellular functions such as migration, proliferation, and apoptosis during inflammation and cancer [1,2,3]. Thus, sphingolipids and their corresponding enzymes are prone to regulate cell fate. Sphingolipid levels are dynamically maintained by the action of Sphingosine kinases (SPHK) 1 and 2, which are the major producers of S1P, by S1P phosphatases (SGPP) 1 and 2 and S1P lyase (SGPL1). An endogenous increase in SPHK 1 levels has been demonstrated in human colon adenomas and mouse models of colon cancer [4,5], and a recent publication showed that intestinal epithelial deletion of SPHK1 prevents colitis-associated cancer development in mice [6]. SPHK inhibitor treatment was shown to lead to a dose-dependent decrease in tumor incidence in mice [7]. Vice versa, SPHK2 knockout mice exhibited an increase in tumor number, size, and load in colitis-associated colon cancer DSS/AOM models [8]. Similarly, SGPP1 was downregulated in gastric cancer tissues, and knockdown of SGPP1 resulted in an increase in the invasion of human gastric carcinoma cell lines [9]. SGPP 2 contributes to ulcerative colitis in mice and humans by promoting mucosal disruption [10]. While S1P may be reversibly degraded by SGPP 1 and 2, SGPL1 alone is able to irreversibly degrade S1P. In a previous study, we found that interference of SGPL1 expression levels augmented a partial re-differentiation of colorectal cancer cells towards normal colon epithelial cells [11]. The cells were inhibited in cell migration and showed a strong upregulation in cell–cell adhesion by increased E-cadherin-actin complexes. Thus, we suggested that manipulation of SGPL1 is associated with the malignancy of already established colon cancer cells. Furthermore, initiation and progression of colon cancer can also be directed by SGPL1 modification [12,13,14]. Our recent study showed a relative and separate contribution of SGPL1 localization in inflammation and carcinogenesis in the pathophysiology of colitis associated colon cancer [15]. A SGPL1 knockout in the immune cell compartment augmented immune cell infiltration, initiating colitis with lesions and subsequent pathological crypt remodeling. Alternatively, a colon-tissue-targeted SGPL1 knockout provoked immediate occurrence of epithelial tumors with a subsequent development of a lower inflammatory but pronounced pro-tumorigenic environment [15]. Survival of these mice was strongly reduced, and the colons had obvious morphological alterations with a significant shortening of colon length, as well as stiffening and thickening of the colon wall. Mice were in bad constitution and suffered from diarrhea, indicating a strong impairment of colon function. Although tumor occurrence was accompanied by a shift in immune cell composition within the malignant regions, the remaining tumor-free colon tissue showed no signs of inflammation or increased immune cell infiltration. In this follow-up study, we performed a detailed immunohistochemical analysis of the colon tissue to investigate the potential occurrence of structural and functional changes of the non-tumorous tissue that might have contributed to the severe phenotype. As detailed in the current investigation, we were able to demonstrate that SGPL1 knockout contributes to cell cycle arrest in the colon stem cell niche while already established colon cancer cells find escape mechanisms from cellular quiescence.

## 2. Results

### 2.1. SGPL1 Knockout Leads to a Shift of Cell Configuration in Colonic Crypts

**Figure 1:**
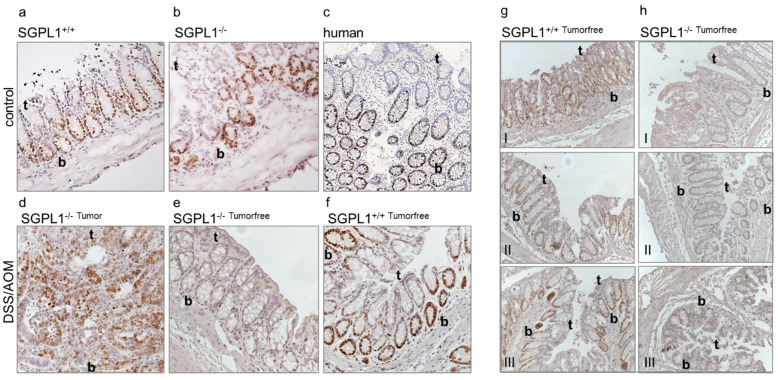
Anti-Ki-67 staining of (**a**) murine colon epithelium (*n* = 5), (**b**) murine SGPL1 knockout colon epithelium (*n* = 5), (**c**) human colon epithelium (*n* = 4), (**d**) murine SGPL1 knockout colon tumor after CAC induction (9 weeks) (*n* = 5), (**e**) murine SGPL1 KO colon epithelium (*n* = 5), (**f**) murine colon epithelium after CAC induction (9 weeks) (*n* = 5 (magnification 40×). Anti-Ki-67 staining (*n* = 3) of (**g**) wildtype and (**h**) SGPL1 knockout colon sections in different locations (I distal colon, II mid colon, III proximal colon); (magnification 20×); CAC = colitis associated colon cancer; in image: t = Crypt tip; b = crypt bottom.

Investigation of non-tumorous colon tissue uncovered a prominent dysregulation of the proliferation marker protein Ki-67 in SGPL1 knockout mice. Under disease-free conditions, Ki-67 positive cells occurred in the crypt bottoms of wildtype and knockout cells similar to the distribution in human colon tissue (Figure 1a–c). During inflammation induced colorectal cancer progression, Ki-67 abundance increased in SGPL1 knockout colon tumors, indicating an increased proliferation rate of cancerous cells (Figure 1d). However, intriguingly, in tumor-free colon tissue of SGPL1 knockout mice in disease, Ki-67 staining completely vanished in all crypt cells (Figure 1e,f).

### 2.2. SGPL1 Knockout Tumors Are Strongly Ki-67 Positive, but the Intestinal Stem Cell Compartment Is Completely Ki-67 Negative

Following our findings, we stained different sections along the colon to see if the phenomenon was present in different locations of the colon. In Figure 1g,h, we show that in the distal, mid, and proximal colon sections of SGPL1 knockout mice in disease, the same Ki-67 negative phenotype was present.

**Figure 2:**
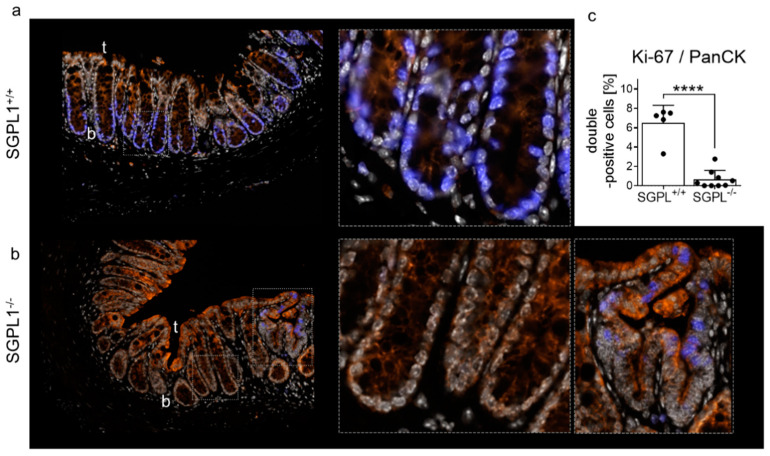
Staining of (**a**) wildtype (SGPL^+/+^) and (**b**) SGPL1 knockout (SGPL^−/−^) colon sections after CAC induction (9 weeks): Dapi (white), Anti-PanCytokeratin (orange), Anti-Ki-67 (blue); (magnification 40× and enhanced crypt bottoms according to frame). (**c**) Quantification of Ki-67/PanCK double positive cells within colon sections without tumors (each black dot represents a single colon section); CAC = colitis associated colon cancer; in image: t = Crypt tip; b = crypt bottom. Significance: **** *p* ≤ 0.0001.

Quantification of Ki-67 staining with multi-fluorescent staining in whole colon sections (including tumor) confirmed the absence of Ki-67 positive cells in SGPL1 knockout colon tissue in disease (Figure 2a–c). In contrast, excitingly, adjacent malformed crypts were Ki-67 positive (Figure 2b, right panel). These Ki-67 positive malformations and also advanced Ki-67-positive tumors directly coexist next to Ki-67 negative crypts in the same colon section (Figure 2b and Figure S1). Quantification of Ki-67 and PanCytokeratin (PanCK) double-positive cells revealed that more than 90% of the Ki-67 positive cells of non-malformed and tumor-free epithelial tissue were lost in SGPL1 knockout colons compared to wildtype during colon cancer progression (Figure 2c).

### 2.3. FOXO3 Expression Is Strongly Enhanced in SGPL1 Knockout Colon Tissue

**Figure 3:**
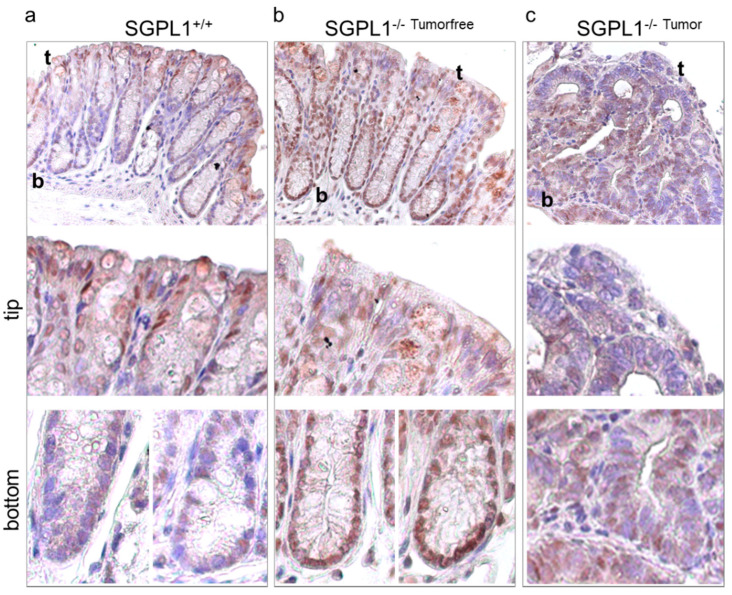
Anti-FOXO3 staining (*n* = 5) of (**a**) wildtype (SGPL1^+/+^) and (**b**) SGPL1 knockout (SGPL^−/−^) colon sections and (**c**) SGPL1 knockout colon tumor sections after CAC induction (9 weeks); (magnification 40× in ‘overview’). CAC = colitis associated colon cancer; in image: t = Crypt tip; b = crypt bottom.

According to literature, Ki-67 negative cells occur in resting quiescent or G0 cells [16]. We, thus, stained the crypts of the wildtype and knockout colons for Forkhead box protein O3 (FOXO3), a negative cell cycle regulator and stemness factor. Under healthy conditions, no difference of FOXO3 staining was observed (Figure S2a,b). Under disease conditions, however, wildtype FOXO3 expression was mainly found at the crypt tips where differentiated cells routinely leave cell cycle and are shed into the lumen (Figure 3a). Crypt bottoms were mainly free of FOXO3 staining. On the contrary, in SGPL1 knockout crypts, a strong FOXO3 staining was present, especially at the crypt bottoms (Figure 3b). Here, all cells were strongly positive for FOXO3, correlating with the negative Ki-67 staining. FOXO3^+^ Ki-67^−^ cells are, thus, very likely in cell cycle arrest. SGPL1 knockout tumor tissue showed areas of low and high FOXO3 staining (Figure 3c).

**Figure 4:**
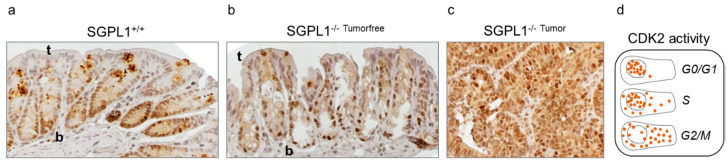
Anti-CDK2 staining (*n* = 5) of (**a**) wildtype (SGPL^+/+^) and (**b**) SGPL1 knockout (SGPL^−/−^) colon sections and (**c**) SGPL1 knockout colon tumor sections after CAC induction (9 weeks); (magnification 40×). (**d**) Overview of CDK2 protein distribution during cell phases (scheme on the basis of [17]); CAC = colitis associated colon cancer; in image: t = Crypt tip; b = crypt bottom.

To further confirm the cell cycle arrest of the crypt bottom cells, we next stained for cyclin-depend-kinase 2 (CDK2) activity, manifesting cell cycle phases according to its protein distribution. While CDK2 staining was heterogeneously distributed in wildtype mice, in knockout cells, CDK2 localization was almost exclusively found in the nuclei of crypt cells (Figure 4). This indicates low CDK2 activity resulting from cells resting in G0- or G1-phase, respectively [17]. In wildtype crypts, CDK2 staining was shared between nuclei and cytoplasm indicating higher CDK2 activity in cell cycle phases S and G2/M.

### 2.4. Transcriptome Analysis of Human SGPL1 Knockout Colorectal Cancer Cells

**Figure 5:**
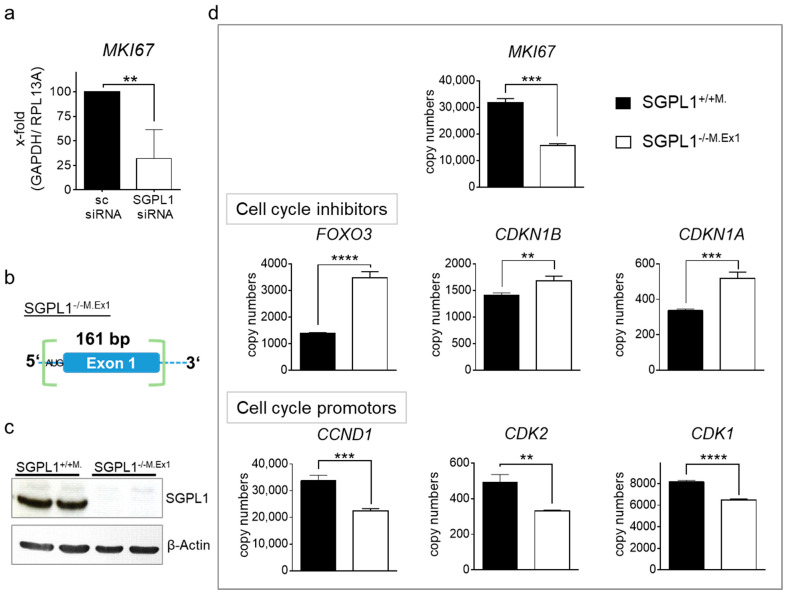
(**a**) MKI67 mRNA expression upon 48 h of scrambled (sc) or SGLP siRNA transfection in DLD-1 cells (*n* = 5); (**b**) schematic overview of sequence modification of stable monoclonal SGPL1-Exon1 knockout DLD-1 cells (SGPL1^−/−M.Ex1^) via CRISPR/Cas9; (**c**) representative Western blot analysis of SGPL1 and β-Actin (*n* = 4); and (**d**) transcriptome analysis (*n* = 3) of SGPL1^+/+M.^ and SGPL1^−/−M.Ex1^. Significances: ** *p* ≤ 0.01; *** *p* ≤ 0.001; **** *p* ≤ 0.0001.

Since we have observed that SGPL1 siRNA treated DLD-1 cells partly re-differentiate into epithelial cells [11], we investigated if SGPL1 siRNA also reduces MKI67 expression. Although, as shown before, proliferation of siRNA treated DLD-1 cells was not different, MKI67 expression faded in siRNA treated cancer cells (Figure 5a). To elucidate this effect in more detail, we generated SGPL1 deficient colorectal cancer cells via CRISPR/Cas9. We designed guide RNAs to exclude a fragment of 161 bp, including Exon 1 and the beforehand start codon, and to induce a frameshift (Figure 5b). These stable monoclonal SGPL1 knockout DLD-1 cells (SGPL1^−/−M.Ex1^) were completely devoid of SGPL1 Exon 1 mRNA and the fully transcribed SGPL1 protein (Figure 5c and Figure S3). S1P abundance was investigated and revealed compensatory mechanisms that prevented S1P accumulation (Figure S3). Transcriptome analysis revealed a decrease in MKI67 copy numbers in stable SGPL1^−/−M.Ex1^ cells compared to empty vector-treated monoclonal SGPL1^+/+M.^ cells (Figure 5d). Gene set enrichment analysis revealed the strongest difference in pathways of apoptosis and cell cycle, including cell cycle transition and cell cycle checkpoint mediators. Herein, copy numbers of cell cycle inhibitors FOXO3, CDKN1B, and CDKN1A were enriched, while cell cycle promoters such as the G1/S checkpoint cyclin D1 (CCND1), as well as CDK2 and CDK1, were reduced in the knockout cells (Figure 5d). As an addition, we found upstream targets of FOXO3, such as HDAC1, HDAC2, and SIRT1, also regulated to favor a FOXO3 increase (Figure S3c).

### 2.5. SGPL1 Knockout Dampens Established PDK1/AKT Signaling in Human Colorectal Cancer Cells but Does Not Induce Cell Cycle Arrest

**Figure 6:**
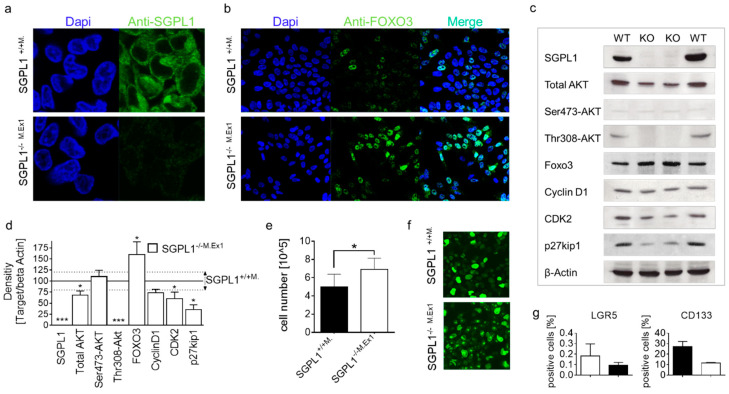
Anti-FOXO3-staining (*n* = 3) of (**a**) SGPL1^+/+M.^ (magnification 60×) and (**b**) stable SGPL1-Exon1 knockout DLD-1 cells (SGPL1^−/−M.Ex1^); (magnification 40×) (**c**) Western blot analysis (*n* = 3), (**d**) protein quantification, (**e**) cell expansion within 72 h (*n* = 8), and (**f**) Anti-Ki-67 staining of SGPL1^+/+^ and SGPL1^−/−M.Ex1^ (*n* = 3); (magnification 40×); (**g**) FACS analysis of LGR5 and CD133 positive cells (*n* = 2). Significances: * *p* ≤ 0.05; *** *p* ≤ 0.001.

In its active form, FOXO3 is present in the nuclei and translocates into the cytoplasm for degradation [18]. In SGPL1^−/−M.Ex1^ cells that were negative for SGPL1 staining (Figure 6a), FOXO3 staining was present in the nuclei of the majority of cells, while only a few cells were positive in wildtype SGPL1^+/+M.^ cells (Figure 6b), indicating a comparable situation to in vivo.

We next performed Western blot analysis to investigate the molecular mechanism of SGPL1 mediated Ki-67 loss based on FOXO3 enhancement (Figure 6c). While total AKT expression was already reduced in SGPL1^−/−M.Ex1^ cells, phosphorylated AKT was completely absent. Especially, Thr308-phopshorylation was missing in knockout cells, indicating a strong reduction of the 3-phosphoinositide dependent kinase-1 (PDK1)/AKT signaling cascade. Confirming the immunofluorescence results, FOXO3 abundance was increased in SGPL1^−/−M.Ex1^ cells. The positive cell cycle regulator CDK2 was marginally reduced; however, cyclin D1 protein abundance was not altered. Additionally, and contrary to expectations, the amount of the cell cycle inhibitor p27kip1 was reduced in SGPL1^−/−M.Ex1^ cells. Proliferation analysis within 72 h additionally revealed an increase of cell division of SGPL1^−/−M.Ex1^ cells (Figure 6e), indicating that cell cycle arrest did not appear in the cancer cells. However, immunofluorescent Ki-67 staining showed numerous large Ki-67 foci in SGPL1^−/−M.Ex1^ cells, indicating a large number of cells being in late G1-phase (Figure 6f). Preliminary analysis of stem cell characteristics of the cells revealed a loss of the stem cell markers LGR5 and CD133 in SGPL1^−/−M.Ex1^ cells (Figure 6g).

## 3. Discussion

**Figure 7:**
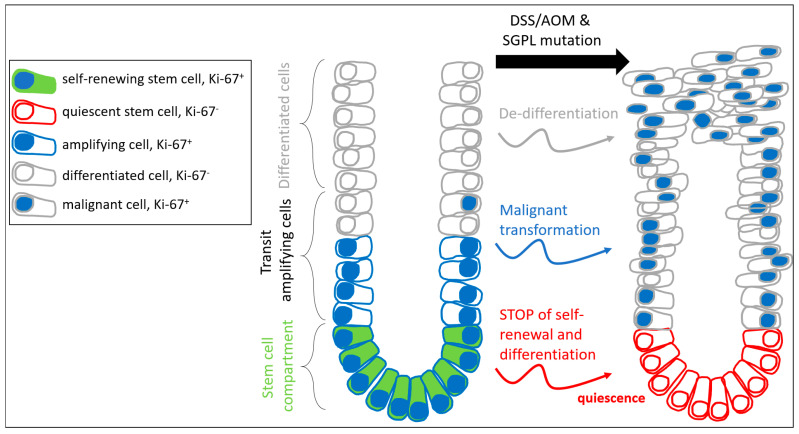
Scheme of S1P lyase mediated cell transformations during colitis-associated colorectal cancer progression.

Ki-67 is an essential component of the perichromosomal layer and coats the condensed chromosomes in mitotic cells. Due to its large size and the distribution of positively charged amino acids, Ki-67 acts as a biological surfactant and prevents aggregation of mitotic chromosomes [19]. Cells in early stages of cell cycle arrest have low levels of Ki-67, which can remain low after re-entering the cell cycle [20]. Cells losing Ki-67 expression do not enter the cell cycle for cell growth and division but develop towards quiescence or senescence. Deeply quiescent and senescent cells do not express Ki-67. As the crypt cells of the SGPL1 knockout mice do not express any Ki-67 under disease conditions, it seems likely that those cells are in cell cycle arrest. Additional indication for the occurrence of cell cycle retention results from the increase of the negative cell cycle regulator FOXO3 and the narrowed activation of the cell cycle promotor CDK2 in these crypt cells. Interestingly, malformed cells and tumor tissue that exist side by side to the residual Ki-67 negative crypts showed prominent Ki-67 abundance. Obviously, cancer cells are able to escape cell cycle arrest and Ki-67 attenuation under SGPL1 knockout (Figure 7).

In G1-phase of the cell cycle, chromosomes de-condense and Ki-67 leaves the perichromosomal layer, resulting in numerous small (early phase) and larger (late phase) Ki-67 positive foci within the nucleus [21]. While the colon cancer wildtype cells (SGPL1^+/+M.^) hardly showed any of these G1-phase characteristics during fluorescence microcopy, the majority of SGPL1^−/−M.Ex1^ cells did assemble numerous large foci (Figure 6f). This might indicate a prolonged retention of these cells in late G1-phase. Cell cycle arrest at one of two critical cell cycle checkpoints before and after DNA replication (G1-S or G2-M checkpoints) allows the cells time to repair their DNA [22]. Thus, under conditions of DNA damage, the phases that go ahead of these checkpoints, G1- and G2-phases, may be prolonged. Recently, it was shown in HEK cells that genetic suppression of SGPL1 by siRNA arrested the cell cycle at the G1-phase and activated cell differentiation [23]. In line with this, we had shown before that SGPL1-mediated Ki-67 loss in SGPL1 siRNA treated DLD-cells undergoes epithelial differentiation by increasing Cytokeratin 20 islets in monolayers and enhancing production of F-actin and E-cadherin [11]. Interestingly, although the SGPL1^−/−M.Ex1^ cells appear to be preserved in the G1-phase, long term proliferation analysis revealed no drawback in cell division rates but rather an advantage over wildtype cells (Figure 6e). Similarly, a study by Cidado et.al. demonstrated that a loss of Ki-67 in DLD-1 cells did not affect proliferation of the cells [24]. However, they determined that Ki-67-loss depletes the cancer stem cell niche. Congruent with this, our preliminary results on stem cell characterization in SGPL1^−/−M.Ex1^ cells showed a trend towards stem cell marker depletion (Figure 6g), indicating a possible contribution of SGPL1 to stemness. This hypothesis is supported by an increase in FOXO3, as FOXO3 increase is described as a stemness suppressor mechanism [25]. It might, thus, be promising to examine the role of SGPL1 in stemness in more detail.

Overexpression of FOXO3 is able to induce a potent G1-arrest [26]. A former study revealed that activation of S1PR1 led to Pi3K/AKT signaling mediated FOXO3 inhibition and promotion of cell survival [27]. According to our transcriptome analysis, DLD-1 cells do not express S1PR1, potentially hindering S1P on inhibiting FOXO3 via this mechanism. However, we observed a distinct decrease in PDK1 mediated AKT phosphorylation and, thus, a dampening of the AKT signaling pathway, of which FOXO3 is a direct target. The absence of AKT mediated FOXO3 phosphorylation hinders cytoplasmatic transport of FOXO3 and degradation, respectively. Additionally, studies also showed that FOXO3 has been increased as a consequence of HDAC1 inhibition in Hek293 cells and ESC [18,19]. In sphingolipid research, it was demonstrated that HDAC1 is decreased in SGPL1 knockout embryonic fibroblasts [28], and we also found in transcriptome analysis of SGPL1^−/−M.Ex1^ cells that the abundance of HDACs was decreased. Therefore, it is possible that decreased HDAC1 expression might contribute to increased FOXO3 expression as a consequence of SGPL1 knockout in SGPL1^−/−M.Ex1^ DLD-1 cells.

The cure for some forms of cancer lies in the G1-arrest of the cell cycle. Emerging evidence indicates that FOXO3 acts as a tumor suppressor in cancer: FOXO3 is commonly inactivated in cancer cell lines either by cytoplasmic sequestration of FOXO3 protein or by mutation of the FOXO3 gene [29]. Its inactivation is, furthermore, associated with the initiation and progression of cancer: FOXO3 knockout mice show increased Ki-67 staining in colon crypts [30] due to increased cell cycle activity, respectively. In experimental studies, overexpression of FOXO3 inhibits the proliferation, tumorigenic potential, and invasiveness of cancer cells, while silencing of FOXO3 results in marked attenuation of protection against tumorigenesis [29]. Many cancers, including breast [27] and skin cancers, [25] have been prevented from proliferating by causing the tumor cells to enter G1 cell cycle arrest, hindering cells from dividing and spreading. In order for the cell to continue cycling through the G1-phase, there must be a high amount of growth factors and a steady rate of protein synthesis, otherwise the cell will move into G0-phase. As many cells in knockout SGPL1^−/−M.Ex1^ DLD-1 were apparently arrested in late G1-phase, the cell cycle inhibitor p27kip1 might have been downregulated by the cancer cells as an escape mechanism from this arrest, to help proceeding through the G1 checkpoint. Thus, it might be that depletion of SGPL1 has a tumor suppressive effect in already established tumor cells.

Mutated colorectal cancer stem cells (CSCs) have the ability to either carry the mutation along with their division to subsequently differentiate or, on the other hand, to repair mutations and proceed or to stop self-renewal and differentiation. Thus, different subpopulations of mutated and non-mutated stem cells, as well as mutated and non-mutated differentiated cells, can occur in the colon. CRISPR/Cas9 mediated DNA damage might induce such repair mechanisms. The potential of CSCs that harbor a complete loss of a larger DNA sequences, rather than a single point mutation, to repair themselves and become non-mutated cells is very unlikely. However, supposedly, CSCs are able to ‘decide’ not to insist on their self-renewing under the SGPL1 knockout mutation to avoid the inescapable process of transferring the mutation into differentiated cells and accomplish potential tumor development. Instead, these cells go into cell cycle arrest and, thus, help to avoid tumor formation. This seems reasonable in a normal environment where mutations occur sporadically in the colon due to environmentally exposure or other events. In the case of a comprehensive initiation of DNA modification, such as the deletion of a DNA sequence through CRISPR/Cas9 in all CSCs, the mechanism of stopping self-renewal, however, can lead to a complete stop of colon tissue renewal. Hence, essential colon functions, depending on its highly regenerative potential, would be doomed to failure, even independently of tumor progression.

## 4. Materials and Methods

### 4.1. Experimental Model of Colitis-Associated Colon Cancer (CAC)

C57BL/6J mice and CreETR2+/− S1PLloxP+/+ mice were bone marrow transplanted to generate a tissue knockout of the SGPL1 and afterwards treated in the DSS/AOM Model for nine weeks as described before [15]. In brief, mice received a single injection (10 mg/kg body weight) of the genotoxic colonic carcinogen azoxymethane (AOM, Sigma-Aldrich, Steinheim, Germany). Chronic colitis was induced by three cycles of 1.5% dextran sodium sulfate (DSS, MP Biomedicals, Eschwege, Germany) in drinking water ad libitum for 5–7 days, followed by two consecutive weeks of normal drinking water. Mice were monitored daily for general condition, body weight, feces, and bleeding. Finally, mice were sacrificed to dissect colon tissue for mRNA and protein analysis as well as for immunohistochemistry.

### 4.2. Immunohistochemistry

Multiple immunofluorescence staining and analysis were performed with the OpalTM 5-Color Fluorescent IHC Kit (Perkin-Elmer, Waltham, MA, USA) according to the manufacturer’s instructions. Prepared paraffin slides were stained with primary antibodies targeting pan Cytokeratin (PCK) (AE1/AE2, Abcam, Berlin, Germany) and Ki-67 (SP6, Abcam). The Vectra^®®^ 3 automated quantitative pathology imaging system (Perkin-Elmer) was used for image acquisition. Bright field immunostaining was performed on prepared paraffin slides with primary antibodies targeting Ki-67 (SP6, Abcam), FOXO3 (D19A7, Cell Signaling Frankfurt, Germany), and CDK2 (E304, Abcam), as described before [31].

### 4.3. Cultivation of DLD-1

The human colorectal cancer cell line DLD-1 was obtained from the American Type Culture Collection (ATCC, Manassas, VA, USA). DLD-1 cells, SGPL1^+/+M.^, and SGPL1^−/−M.Ex1^ cells were grown in RPMI 1640 Medium (Gibco, Waltham, MA, USA), supplemented with 10% FCS, 100 IU/mL penicillin, and 100 μg/mL streptomycin (Sigma-Aldrich). Cells had been tested negative for mycoplasm contamination. All cell lines were cultured in an incubator with 5% CO_2_ at 37 °C.

### 4.4. Transfection with siRNA

DLD-1 cells were transfected using DharmaFECT 1 Transfection Reagent with the On-TARGETplus human SGPL1 siRNA SMARTpool (L-008747, Horizon, Cambridge, UK) or scramble control siRNA (SIC001, Sigma-Aldrich), as described before [11].

### 4.5. CRISPR/Cas9

Guide RNAs for SGPL1 gene silencing were designed (TCTGGCGAATCTAGGCGGGC, GAGACAAATGCCTTGGAACC) and integrated into the pSpCas9(BB)-2A-Puro (PX459) V2.0 (Addgene, Teddington, UK) backbone vector. The modified vector and the empty vector as a control were amplified in DH5alpha cells and isolated with the QIAprepSpin Miniprep Kit(250) (Qiagen, Hilden, Germany). The plasmids were sent for Sanger sequencing to confirm successful cloning. The plasmids were then transfected each into DLD-1 cells with DharmaFECT 1 Transfection Reagent (Horizon), and positive selection was performed with puromycin. Single cell culture was performed with conditioned medium and cloning cylinders. Single cell clones were cultured for 3 weeks to reach sufficient cell numbers. DNA was isolated with the peqGOLD MicroSpin Tissue DNA Kit (VWR, Darmstadt, Germany), and successful SGPL1 gene disruption was confirmed by sequencing with FW Primer TTTTGATTCGCTGGTCTGGG and RV Primer CCAAAAGCAAGCATCAGAGGT. CRISPR/CAS9 led to the loss of Exon 1, followed by a frame shift in the SGPL1 gene and no detectable Exon1 mRNA or protein amounts, respectively. Knockout cells are referred to as SGPL1^−/−M.Ex1^.

### 4.6. RNA-Sequencing

Whole transcriptome RNA sequencing was performed at the Max Planck Institute for Heart and Lung Research Bad Nauheim, as described before [32].

### 4.7. Flow Cytometry

Flow Cytometry for the detection of stem cell markers on DLD-1 cells was performed as described before [33]. Anti-LGR5 (ab75732, Abcam) and PE-anti CD133 (Clone 7, Biolegend, San Diego, CA, USA) were used.

### 4.8. Liquid Chromatography Tandem Mass Spectrometry

Quantification of S1P was performed by high-performance liquid chromatography tandem mass spectrometry, as described before [34].

### 4.9. Western Blot

For Western blot analysis, pelleted cells were lysed, protein concentration was determined, and whole cell extracts were used for SDS-Page and semi-dry Western blotting, with subsequent detection and quantification as described before in Ref. [2] Target detection was performed with antibodies against SGPL1 (HPA021125; Atlas Antibodies, Bromma, Sweden,), panAKT (C67E7), Phospho-Ser472-AKT (D9E), Phospho-Thr308-AKT (D25E6), FOXO3 (D19A7), p27kip1a (D69C12), Cyclin D1 (E3P5S) from Cell Signaling, CDK2 (E304, Abcam), and β-Actin (A5441, Sigma-Aldrich) and according secondary antibodies, anti-rabbit IgG or anti-mouse IgG (GE Healthcare, Little Chalfont, UK).

### 4.10. Immunofluorescence Staining

Cells were grown on tissue-treated 8-well chambered cover slides (Ibidi, Martinsried, Germany) and fixed as described before in Ref. [11] Primary SGPL1 antibody SGPL1 (HPA021125, Atlas Antibodies), FOXO3a (D19A7, Cell Signaling), Ki-67 (SP6, Abcam), as well as secondary anti-rabbit IgG (GE Healthcare) and DAPI (4′,6-Diamidine-2′-phenylindole dihydrochloride, Roche Diagnostics, Mannheim, Germany), were used. Confocal laser scanning microscopy was performed with a Zeiss LSM510 Meta system equipped with an inverted Observer Z1 microscope and a Plan-Apochromat 63×/1.4 or 40× oil immersion or 20× objective (Carl Zeiss MicroImaging GmbH, Göttingen, Germany).

### 4.11. RNA Isolation and Real-Time PCR

Total RNA was extracted using the peqGOLD Total RNA Kit (VWR) as recommended by the manufacturer. RNA concentration was adjusted by the Nano-Drop 1000 analyzer (Thermo Fisher Scientific, Waltham, MA, USA) for the synthesis of cDNA using the high-capacity cDNA reverse transcription kit (Thermo Fisher Scientific). TaqMan^®®^ gene expression assays (Thermo Fisher Scientific) were applied for all genes of interest and for the housekeeping genes GAPDH and FBXO38 (Primer Design, Southampton, UK). The Precision FAST Mastermix (Primer Design) was used, and quantitative real-time PCR was run according to manufactures’ recommendations (7500 Fast Real-Time PCR System, Applied Biosystems). Data were evaluated using the mean of the two housekeeping genes as a reference.

### 4.12. Statistics

GraphPad Prism 7.0 (La Jolla, CA, USA) software was used to enter data, display graphs, and perform statistics by unpaired Student’s t tests, if not otherwise indicated. Data are represented as means ± SD and significant values representing *p*-values of ≤0.05/≤0.01/≤0.001/≤0.0001 are symbolized as asterisks (*/**/***/****).

## Data Availability

The data presented in this study are available on request from the corresponding authors.

## Supplementary Materials

The following are available online at https://www.mdpi.com/article/10.3390/ijms22115682/s1.

## References

[1] Duan R.D., Nilsson A. (2009). Metabolism of sphingolipids in the gut and its relation to inflammation and cancer development. Prog. Lipid Res..

[2] Schwiebs A., Friesen O., Katzy E., Ferreiros N., Pfeilschifter J.M., Radeke H.H. (2016). Activation-Induced cell death of dendritic cells is dependent on sphingosine kinase 1. Front. Pharmacol..

[3] Arlt O., Schwiebs A., Japtok L., Ruger K., Katzy E., Kleuser B., Radeke H.H. (2014). Sphingosine-1-phosphate modulates dendritic cell function: Focus on non-migratory effects in vitro and in vivo. Cell Physiol. Biochem..

[4] Kawamori T., Kaneshiro T., Okumura M., Maalouf S., Uflacker A., Bielawski J., Hannun Y.A., Obeid L.M. (2009). Role for sphingosine kinase 1 in colon carcinogenesis. FASEB J..

[5] Liu S.Q., Su Y.J., Qin M.B., Mao Y.B., Huang J.A., Tang G.D. (2013). Sphingosine kinase 1 promotes tumor progression and confers malignancy phenotypes of colon cancer by regulating the focal adhesion kinase pathway and adhesion molecules. Int. J. Oncol..

[6] Park S.B., Choi B.I., Lee B.J., Kim N.J., Jeong Y.A., Joo M.K., Kim H.J., Park J.J., Kim J.S., Noh Y.S. (2020). Intestinal epithelial deletion of sphk1 prevents colitis-associated cancer development by inhibition of epithelial stat3 activation. Dig. Dis. Sci..

[7] Chumanevich A.A., Poudyal D., Cui X., Davis T., Wood P.A., Smith C.D., Hofseth L.J. (2010). Suppression of colitis-driven colon cancer in mice by a novel small molecule inhibitor of sphingosine kinase. Carcinogenesis.

[8] Liang J., Nagahashi M., Kim E.Y., Harikumar K.B., Yamada A., Huang W.C., Hait N.C., Allegood J.C., Price M.M., Avni D. (2013). Sphingosine-1-phosphate links persistent STAT3 activation, chronic intestinal inflammation, and development of colitis-associated cancer. Cancer Cell.

[9] Gao X.Y., Li L., Wang X.H., Wen X.Z., Ji K., Ye L., Cai J., Jiang W.G., Ji J.F. (2015). Inhibition of sphingosine-1-phosphate phosphatase 1 promotes cancer cells migration in gastric cancer: Clinical implications. Oncol. Rep..

[10] Huang W.C., Liang J., Nagahashi M., Avni D., Yamada A., Maceyka M., Wolen A.R., Kordula T., Milstien S., Takabe K. (2016). Sphingosine-1-phosphate phosphatase 2 promotes disruption of mucosal integrity, and contributes to ulcerative colitis in mice and humans. FASEB J..

[11] Faqar-Uz-Zaman W.F., Schmidt K.G., Thomas D., Pfeilschifter J.M., Radeke H.H., Schwiebs A. (2021). S1P lyase siRNA dampens malignancy of DLD-1 colorectal cancer cells. Lipids.

[12] Oskouian B., Sooriyakumaran P., Borowsky A.D., Crans A., Dillard-Telm L., Tam Y.Y., Bandhuvula P., Saba J.D. (2006). Sphingosine-1-phosphate lyase potentiates apoptosis via p53- and p38-dependent pathways and is down-regulated in colon cancer. Proc. Natl. Acad. Sci. USA.

[13] Takahashi K., Fujiya M., Konishi H., Murakami Y., Iwama T., Sasaki T., Kunogi T., Sakatani A., Ando K., Ueno N. (2020). Heterogenous nuclear ribonucleoprotein H1 promotes colorectal cancer progression through the stabilization of mRNA of sphingosine-1-phosphate lyase 1. Int. J. Mol. Sci..

[14] Degagne E., Pandurangan A., Bandhuvula P., Kumar A., Eltanawy A., Zhang M., Yoshinaga Y., Nefedov M., de Jong P.J., Fong L.G. (2014). Sphingosine-1-phosphate lyase downregulation promotes colon carcinogenesis through STAT3-activated microRNAs. J. Clin. Investig..

[15] Schwiebs A., Herrero San Juan M., Schmidt K.G., Wiercinska E., Anlauf M., Ottenlinger F., Thomas D., Elwakeel E., Weigert A., Farin H.F. (2019). Cancer-induced inflammation and inflammation-induced cancer in colon: A role for S1P lyase. Oncogene.

[16] Booth D.G., Earnshaw W.C. (2017). Ki-67 and the chromosome periphery compartment in mitosis. Trends Cell Biol..

[17] Spencer S.L., Cappell S.D., Tsai F.C., Overton K.W., Wang C.L., Meyer T. (2013). The proliferation-quiescence decision is controlled by a bifurcation in CDK2 activity at mitotic exit. Cell.

[18] Huang H., Tindall D.J. (2007). Dynamic FoxO transcription factors. J. Cell Sci..

[19] Cuylen S., Blaukopf C., Politi A.Z., Muller-Reichert T., Neumann B., Poser I., Ellenberg J., Hyman A.A., Gerlich D.W. (2016). Ki-67 acts as a biological surfactant to disperse mitotic chromosomes. Nature.

[20] Sobecki M., Mrouj K., Colinge J., Gerbe F., Jay P., Krasinska L., Dulic V., Fisher D. (2017). Cell-Cycle regulation accounts for variability in Ki-67 expression levels. Cancer Res..

[21] Matheson T.D., Kaufman P.D. (2017). The p150N domain of chromatin assembly factor-1 regulates Ki-67 accumulation on the mitotic perichromosomal layer. Mol. Biol. Cell.

[22] Zhou B.B., Elledge S.J. (2000). The DNA damage response: Putting checkpoints in perspective. Nature.

[23] Jeon S., Song J., Lee D., Kim G.T., Park S.H., Shin D.Y., Shin K.O., Park K., Shim S.M., Park T.S. (2020). Inhibition of sphingosine 1-phosphate lyase activates human keratinocyte differentiation and attenuates psoriasis in mice. J. Lipid Res..

[24] Cidado J., Wong H.Y., Rosen D.M., Cimino-Mathews A., Garay J.P., Fessler A.G., Rasheed Z.A., Hicks J., Cochran R.L., Croessmann S. (2016). Ki-67 is required for maintenance of cancer stem cells but not cell proliferation. Oncotarget.

[25] Liu Y. (2019). Targeting the non-canonical AKT-FOXO3a axis: A potential therapeutic strategy for oral squamous cell carcinoma. EBioMedicine.

[26] Medema R.H., Kops G.J., Bos J.L., Burgering B.M. (2000). AFX-like Forkhead transcription factors mediate cell-cycle regulation by Ras and PKB through p27kip1. Nature.

[27] Safarian F., Khallaghi B., Ahmadiani A., Dargahi L. (2015). Activation of S1P(1) receptor regulates PI3K/Akt/FoxO3a pathway in response to oxidative stress in PC12 cells. J. Mol. Neurosci..

[28] Ihlefeld K., Claas R.F., Koch A., Pfeilschifter J.M., Meyer Zu Heringdorf D. (2012). Evidence for a link between histone deacetylation and Ca(2)+ homoeostasis in sphingosine-1-phosphate lyase-deficient fibroblasts. Biochem. J..

[29] Liu Y., Ao X., Ding W., Ponnusamy M., Wu W., Hao X., Yu W., Wang Y., Li P., Wang J. (2018). Critical role of FOXO3a in carcinogenesis. Mol. Cancer.

[30] Penrose H.M., Cable C., Heller S., Ungerleider N., Nakhoul H., Baddoo M., Hartono A.B., Lee S.B., Burow M.E., Flemington E.F. (2019). Loss of forkhead box O3 facilitates inflammatory colon cancer: Transcriptome profiling of the immune landscape and novel targets. Cell Mol. Gastroenterol. Hepatol..

[31] Huhn M., Juan M.H.S., Melcher B., Dreis C., Schmidt K.G., Schwiebs A., Collins J., Pfeilschifter J.M., Vieth M., Stein J. (2020). Inflammation-Induced mucosal KYNU expression identifies human ileal crohn’s disease. J. Clin. Med..

[32] Wang L., Wang S., Shi Y., Li R., Gunther S., Ong Y.T., Potente M., Yuan Z., Liu E., Offermanns S. (2020). YAP and TAZ protect against white adipocyte cell death during obesity. Nat. Commun..

[33] Dreis C., Ottenlinger F.M., Putyrski M., Ernst A., Huhn M., Schmidt K.G., Pfeilschifter J.M., Radeke H.H. (2019). Tissue cytokine IL-33 modulates the cytotoxic CD8 T lymphocyte activity during nutrient deprivation by regulation of lineage-specific differentiation programs. Front. Immunol..

[34] Schwiebs A., Thomas D., Kleuser B., Pfeilschifter J.M., Radeke H.H. (2017). Nuclear translocation of SGPP-1 and decrease of SGPL-1 activity contribute to sphingolipid rheostat regulation of inflammatory dendritic cells. Mediators Inflamm..

