# Supplementary material for: S1P Lyase Regulates Intestinal Stem Cell Quiescence via Ki-67 and FOXO3
Source: Int J Mol Sci. 2021 May 26;22(11):5682. doi: 10.3390/ijms22115682 (PMC8198365; doi:10.3390/ijms22115682)
Supplement: Supplementary file 1 [file ijms-22-05682-s001.zip › ijms-1220275-supplementary.pdf]

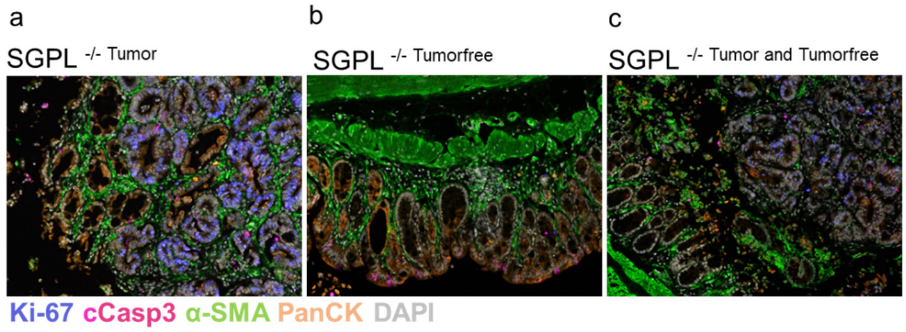

Figure S1. Multi-color staining of (**a**) SGPL1$^{-/-}$ tumor tissue, (**b**) SGPL1 $^{-/-}$ tumor-free tissue and (**c**) SGPL1$^{-/-}$ tissue with tumor-free and tumor areas (magnification 40×).

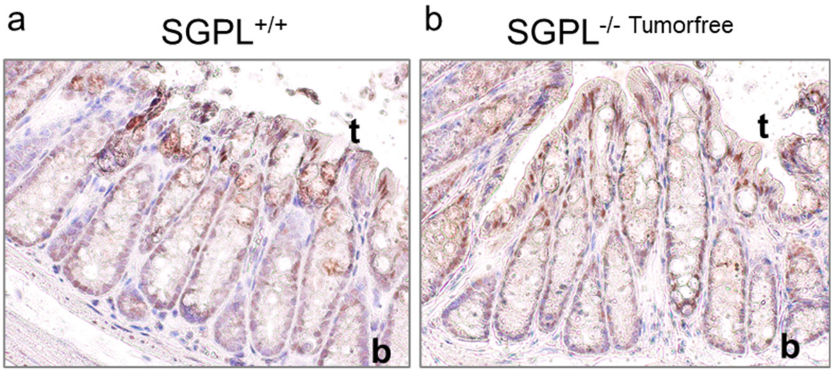

Figure S2. Anti-FOXO3a staining of a) wildtype (SGPL$^{+/+}$) and b) SGPL1 knockout (SGPL$^{-/-}$) colon sections (magnification 40×); in image: t = crypt tip; b = crypt bottom.

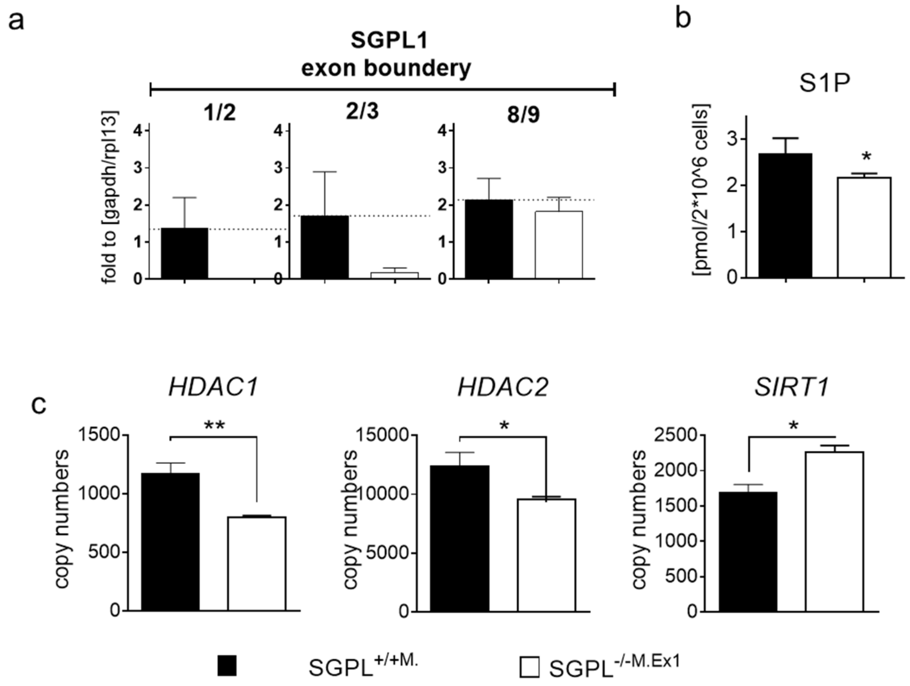

Figure S3. (**a**) SGPL1 mRNA expression targeted by different primers for exon boundaries 1/2, 2/3 and 8/9; (**b**) S1P quantification via MS/MS, (**d**) transcriptome analysis of SGPL1$^{+/+M.}$ and SGPL1$^{-/-M.Ex1}$ cells.
